# The role of concurrent chemotherapy for stage II nasopharyngeal carcinoma in the intensity-modulated radiotherapy era: A systematic review and meta-analysis

**DOI:** 10.1371/journal.pone.0194733

**Published:** 2018-03-22

**Authors:** Fang Liu, Tao Jin, Lei Liu, Zhongzheng Xiang, Ruonan Yan, Hui Yang

**Affiliations:** 1 The Department of Medical Oncology, Cancer Center, State Key Laboratory of Biotherapy, West China Hospital, Sichuan University, Chengdu, Sichuan, China; 2 Department of Urology, Institute of Urology, West China Hospital, Sichuan University, Chengdu, Sichuan, China; 3 Department of Otolaryngology-Head and Neck Surgery, West China Hospital, Sichuan University, Chengdu, Sichuan, China; George Washington University Milken Institute of Public Health, UNITED STATES

## Abstract

**Objectives:**

To compare clinical outcomes of concurrent chemoradiotherapy (CCRT) with those of radiotherapy alone for stage II nasopharyngeal carcinoma in the intensity-modulated radiotherapy (IMRT) era.

**Materials and methods:**

We comprehensively searched PubMed, Embase, and the Cochrane Library to identify eligible studies. Overall survival (OS), progression-free survival (PFS), distant metastasis-free survival (DMFS), locoregional recurrence-free survival (LRRFS) with hazard ratios (HRs), and toxicities with odd ratios (ORs) were analyzed.

**Results:**

A total of seven studies met the criteria, with 1302 patients who were treated with IMRT alone or IMRT plus concurrent chemotherapy. No significant survival benefit was shown by CCRT regardless of OS (HR = 1.17, 95% CI 0.73–1.89, P = 0.508), PFS (HR = 0.76, 95% CI 0.38–1.50, P = 0.430), DMFS (HR = 0.89, 95% CI 0.33–2.41, P = 0.816), or LRRFS (HR = 1.03, 95% CI 0.95–1.12, P = 0.498). Additionally, CCRT notably increased the risk of acute grade 3–4 leukopenia (OR = 4.432, 95% CI 2.195–8.952, P < 0.001), compared to IMRT alone.

**Conclusion:**

Adding concurrent chemotherapy to IMRT led to no survival benefit and increased acute toxicity reactions for stage II nasopharyngeal carcinoma.

## Introduction

Nasopharyngeal carcinoma (NPC) is quite rare in Europe and the United States but relatively endemic in Southeast Asia, Southern China, the Arctic, and North Africa, especially in Southern China[1, 2]. Radiotherapy is the primary and only curative treatment modality. Additionally, sequential and/or concurrent chemotherapy is widely applied for the treatment of NPC for its chemosensitivity. As is well known, concurrent chemoradiotherapy (CCRT) with/without adjuvant chemotherapy is recommended for locoregionally advanced NPC cases, and radiotherapy alone is suggested for stage I NPC patients. With regard to stage II NPC, CCRT is more acceptable[3, 4]. A phase III randomized trial[5] by Chen et al. demonstrated that adding concurrent chemotherapy to two-dimensional radiotherapy (2D-RT) significantly improved overall survival (OS) in stage II (the Chinese 1992 staging system) NPC, predominantly through a decrease in distant failures. In the context of conventional radiotherapy, many other trials[6, 7] have also determined that CCRT can improve survival for stage II NPC compared with radiotherapy alone. Recently, intensity-modulated radiotherapy (IMRT), an advanced form of conventional radiotherapy and a great stride in the management of NPC, has been widely used clinically. Compared to conventional 2D-RT, this technique offers a more satisfactory balance between target dose coverage and the sparing of adjacent organs at risk. As a number of studies have confirmed, IMRT is superior to conventional radiotherapy in local control, progression-free survival (PFS), and even OS[8–11]. Thus, a crucial question is whether stage II NPC patients can still obtain a significant survival benefit from concurrent chemotherapy in the IMRT era. Additionally, some studies have reported satisfactory therapeutic effects in stage II NPC patients treated with IMRT alone[12, 13].

A recent meta-analysis[14] explored the value of chemoradiotherapy in stage II NPC compared to radiotherapy alone. However, patients treated with various neoadjuvant chemotherapy or adjuvant chemotherapy combined with CCRT were included. The real role of adding concurrent chemotherapy to IMRT for NPC patients remains unclear. Hence, we performed this study to compare the clinical treatment outcomes and toxicities of pure CCRT with those of IMRT alone for stage II NPC patients, with the hope of providing valuable evidence for treatment guidelines and suggestions for future trials.

## Material and methods

### Literature search strategy

This systematic review and meta-analysis was conducted according to Preferred Reporting Items for Systematic Review and Meta-analysis (PRISMA)[15]. The electronic databases Embase, PubMed, and the Cochrane Library were comprehensively searched for all relevant studies without restrictions to language or region before June 13, 2017. The following search terms and their combinations were used: (nasopharyngeal OR nasopharynx) AND (carcinoma OR cancer OR neoplasm OR tumor OR malignant OR malignancy) AND (radiotherapy) AND (chemotherapy). To ensure a comprehensive review, we also screened the citation lists of all included articles.

### Selection criteria

All eligible trials had to meet the following predefined criteria: (1) studies that compared IMRT plus concurrent chemotherapy versus IMRT alone in stage II NPC patients; (2) included patients were previously untreated with histologically proven NPC; (3) patients who received neoadjuvant chemotherapy or adjuvant chemotherapy were excluded; (4) at least one of the following terms could be acquired from the paper directly or indirectly using Tierney’s Methods[16]: time-to-event data including OS, PFS, distant metastasis-free survival (DMFS), and locoregional recurrence-free survival (LRRFS), and instances of grade 3–4 adverse events; and (5) commentaries, editorials, reviews, case reports, and letters to editors were excluded.

### Data extraction

Two investigators independently extracted relevant characteristics from all included studies using a standard extraction form. For each individual study, we summarized the data including first author, publication year, study design, inclusion period, region where research was conducted, number of patients, histologic type (WHO criteria), staging system and detailed stage data, follow-up duration, treatment protocols, time-to-event data (OS, PFS, DMFS, LRRFS), and instances of acute grade 3–4 adverse events. Any discrepancies were resolved by consulting with a third researcher. If necessary, the study authors were contacted via e-mail.

The primary outcomes were OS, PFS, DMFS, and LRRFS. OS was defined as the time from diagnosis until death or the latest day known to be alive. The duration of time to distant metastasis or recurrence was counted from the initiation of treatment to treatment failure. The secondary end points were the rates of acute grade 3–4 toxicity reactions including hematological events (anemia, leukopenia, neutropenia, thrombocytopenia) and non-hematological events (mucositis and gastrointestinal).

### Quality assessment and data analysis

The Cochrane risk of bias tool[17] and the modified Newcastle-Ottawa scale[18] were used to appraise the methodological quality of included randomized controlled trials and retrospective studies, respectively. The quality of each retrospective study was scored ranging from 0 to 9, and studies with scores ≥ 6 were considered high-quality. Furthermore, according to the criteria published by Oxford Centre for Evidence-Based Medicine[19], the levels of evidence for each included studies were evaluated.

Statistical analyses were performed using STATA 14 (STATA Corporation, College Station, TX, USA). All time-to-event data (OS, PFS, DMFS, LRRFS) were expressed as hazard ratios (HRs) and 95% confidence intervals (CIs). Odd ratios (ORs) with 95% CIs were used as summary statistics for toxicities. If the 95% CI did not include the value 1 with P < 0.05, the estimate of the outcome was considered statistically significant. An observed HR or OR <1 signified that patients treated with CCRT had survival benefits or sustained less toxicities. Statistical heterogeneity across studies was evaluated using the Cochrane Q test and the I^2^ statistic[20–22]. Heterogeneity was defined as when the P value of the Cochrane Q test was < 0.10 or the I^2^ value was > 50%. If P > 0.10 and I^2^ < 50%, a fixed-effects model was applied for analysis. If not, a random-effects model was used.

For sensitivity analysis, we excluded several trials each time according to different criteria and analyzed the remaining trials to assess the stability of the results. Publication bias was evaluated using Begg’s and Egger’s tests, P > 0.1 was considered no potential publication bias[23, 24].

## Results

### Search results and characteristics of studies

**Fig 1:**
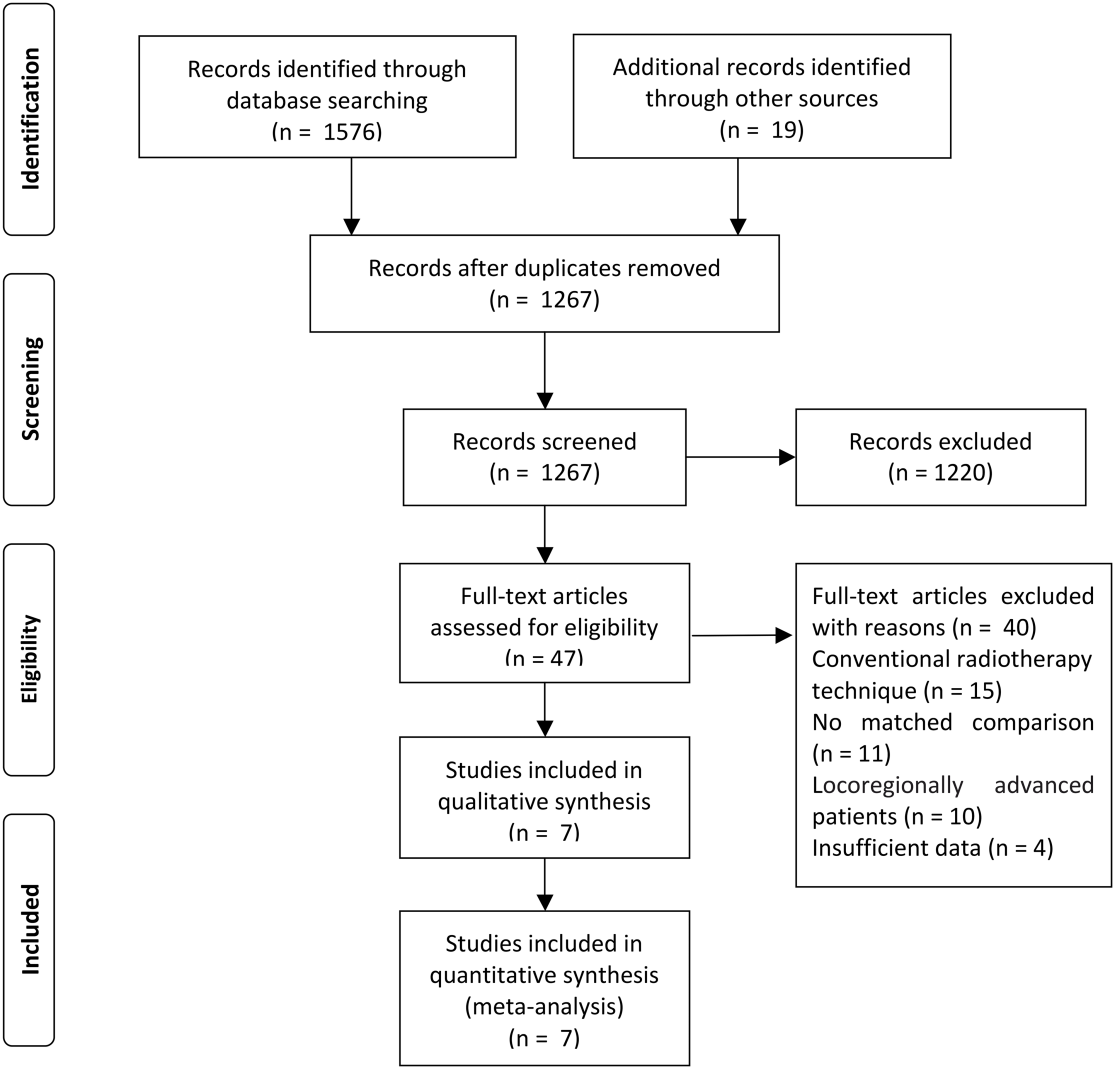
Flow chart showing inclusion and exclusion of trials.

A total of 1595 studies were identified from the databases and references. After excluding 328 duplicate publications, 1220 non-relevant studies were discarded by screening their titles and/or abstracts. Of the 47 full-text articles assessed for eligibility, 11 were abandoned for no matched comparison, 15 for using non-IMRT technique, 10 for including patients with locoregionally advanced NPC, and 4 for lack of time-to-event data (OS, PFS, DMFS, LRRFS) or instances of adverse events, as we predefined. Consequently, 7 trials[25–31] fulfilled the inclusion criteria, and the flow diagram is presented in Fig 1.

Of 1302 total patients included in this study, 716 received CCRT and 586 received IMRT alone. All seven studies were performed in China. Apart from a single randomized controlled trial[30], six of the seven trials were retrospective studies. All studies recruited stage II NPC patients except for two studies that also included a small fraction of stage III patients (T3N0M0, 18.0%)[27] and stage I patients (T1N0M0, 23.2%)[31]. The general quality of the seven studies was evaluated, and four were classified as high-quality. Moreover, four studies reached evidence level 2b. The baseline characteristics of the included studies are summarized in Table 1.

**Table 1:** Main characteristics of all the included studies.

Study	Inclusion Period	Study design	Patients (treatment/control)	Female% (treatment/control)	Median age (treatment/control)	Histology (WHO classification)			Clinical stage	Median follow-up time(range),mo.	Concurrent chemotherapy	IMRT	High quality	Level of evidence[19]
						I	II	III						
Ng/2015	2004–2012	R	210(124/86)	NA	NA(48.0/52.2)	NA			AJCC 7th edition II	49.2(NA)	57: cisplatin 40 mg/m^2^, qw; 59: cisplatin 100 mg/ m^2^, q3w; 8: carboplatin	70Gy/33f	No	4
Chen/2016	2007–2014	R	122(80/42)	32.0(28.8/38.1)	NA		14	108	AJCC 7th edition II	56.0(9.0–100.0)	2–3 × q3w cisplatin 80–100 mg/m^2^	68-70Gy/30-31f for PGTVnx and PGTVnd, 60-66Gy/30-31f for PCTV1, 50-56Gy/30-31f for PCTV2	Yes	2b
Zhang/2015	2003–2013	R	482(241/241)	25.5(25.3/25.7)	NA(47/46)		16	466	AJCC 7th edition II +T3N0M0(18.0%)	47.6(10.0–138.0)/50.7(10.9–138.0)	2–3 × q3w cisplatin- or nedaplatin-based regimen 80–100 mg/m^2^;or cisplatin- or nedaplatin-based regimen 30–40 mg/m^2^, qw; or docetaxel-based regimen 20–30 mg/m^2^, qw	68Gy/30f for PGTVnx, 60-66Gy/30f for PGTVnd, 60Gy/30f for PCTV1, 54Gy/30f for PCTV2	Yes	2b
Su/2016	2005–2010	R	249(143/106)	28.5(30.8/25.5)	NA		13	236	AJCC 7th edition II	59.4(4.0–115.7)	123: platinum single-agent (qw or q3w),20: paclitaxel, PF, or TP	66-70Gy/25-30f for PGTVnx, 60-64Gy/25-30f for PGTVnd, 55-62Gy/25-30f for PCTV1, 42-54Gy/25-30f for PCTV2	Yes	4
Xu/2015	2009–2011	R	86(43/43)	26.7(25.6/27.9)	50(50/51)	NA			AJCC 6th edition II	37.4(4.8–66.2)	cisplatin 40 mg/m^2^, qw	66Gy/30f for PGTVnx and PGTVnd, 60Gy/30f for PCTV1, 54Gy/30f for PCTV2	Yes	2b
Yi/2015	2010–2012	RCT	84(41/43)	NA	NA	NA			AJCC 7th edition II	38.0(NA)	cisplatin 40 mg/m^2^, qw	NA	No	2b
Luo/2014	2006–2010	R	69(44/25)	44.9(NA)	42(NA)		49	20	AJCC 6th edition II + T1N0M0(23.2%)	34.0(12.0–64.0)	cisplatin 80–100 mg/m^2^, q3w	68-72Gy/30-33f for PGTVnx, 66-70Gy/30-33f for PGTVnd, 60-63Gy/30-33f for PCTV1, 50.4-56Gy/28f for PCTV2	No	4

Abbreviations: R, retrospective; RCT, randomized controlled trial; AJCC, American Joint Committee on Cancer; WHO, World Health Organization (WHO classification: type I, squamous cell carcinoma; type II, nonkeratinizing carcinoma; type III, undifferentiated carcinoma); NA, not available; mo., months; IMRT, intensity-modulated radiotherapy; qw, weekly; q3w, every 3 weeks; PF, cisplatin combined with 5-fluorouracil; TP, docetaxel combined with cisplatin; f, fraction; GTV, gross tumor volume; CTV, clinical target volume.

### Survival outcome

**Fig 2:**
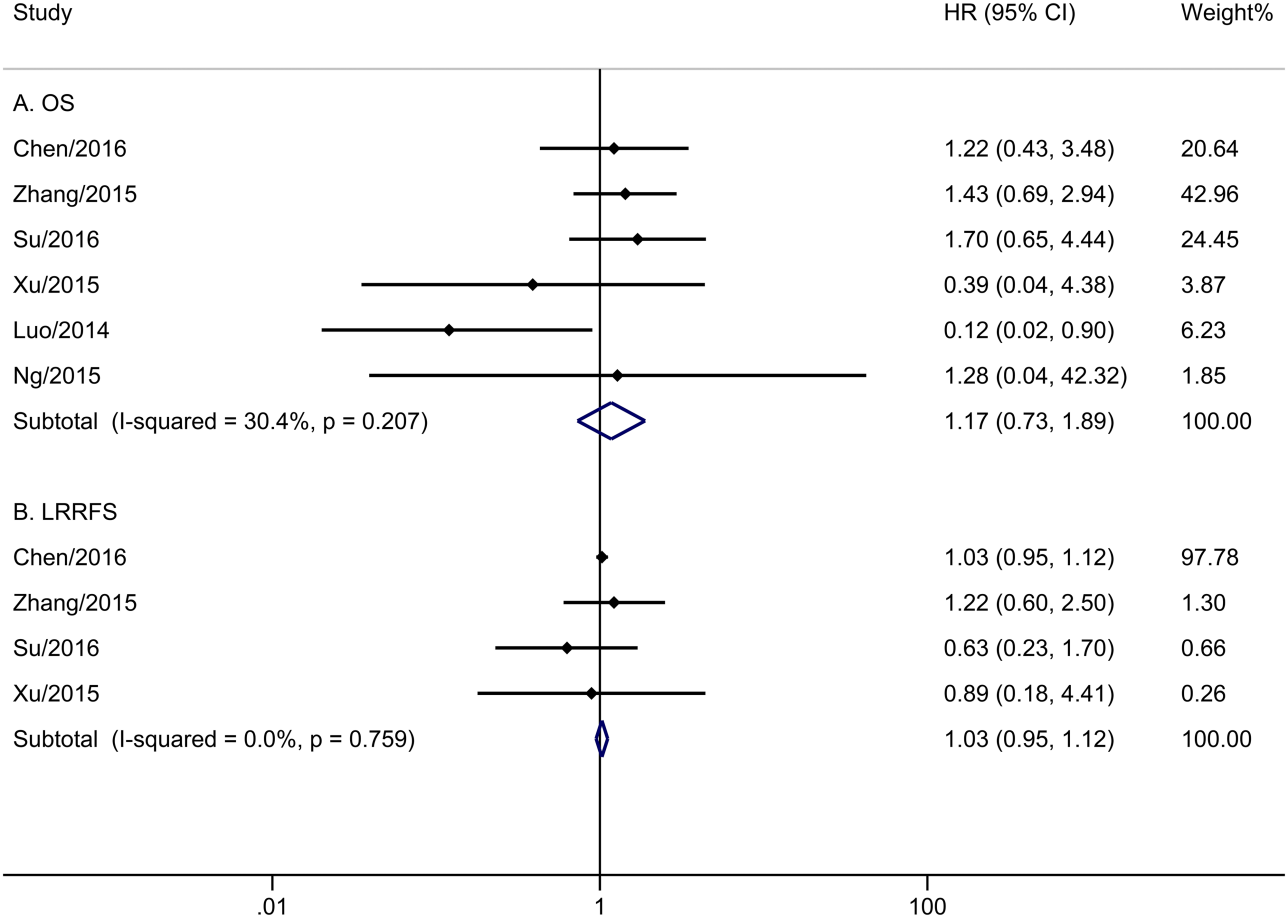
Forest plot and meta-analysis of OS (A) and LRRFS (B).

**Fig 3:**
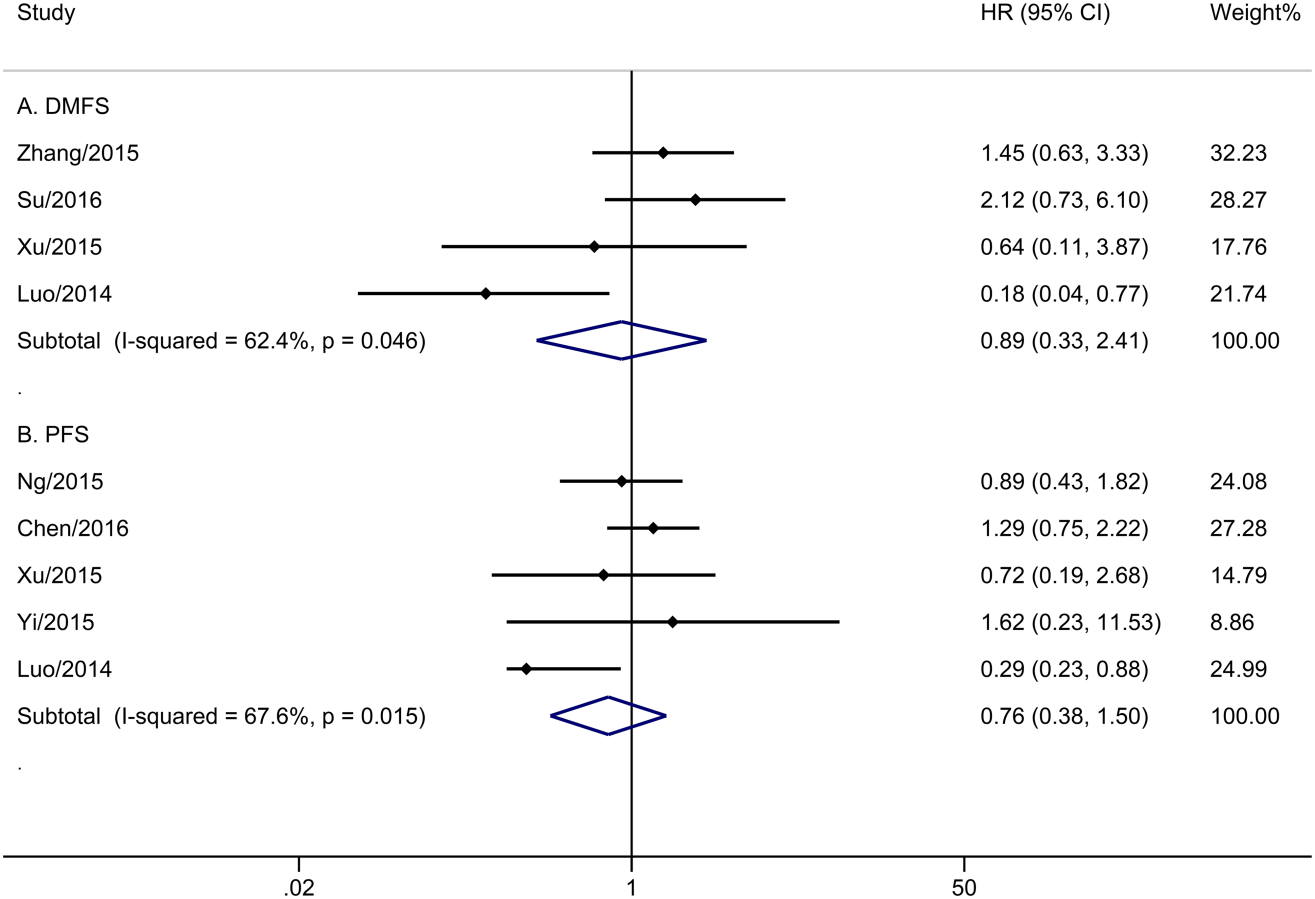
Forest plot and meta-analysis of DMFS (A) and PFS (B).

The meta-analysis of OS was based on six trials with 1218 patients. No obvious heterogeneity was observed among these trials (P = 0.207, I^2^ = 30.4%). Analysis by a fixed-effects model showed that the CCRT group did not have improved OS compared with the IMRT alone group (HR = 1.17, 95% CI 0.73–1.89, P = 0.508; Fig 2A). Five trials with 571 patients reported PFS (Fig 3B). The merge HR was 0.76 (95% CI 0.38–1.50, P = 0.430; heterogeneity: P = 0.015, I^2^ = 67.6%), indicating that there was no significant difference in PFS between the two groups. Four studies with 886 cases reported DMFS. Fig 3A shows the pooled results. Unfortunately, stage II NPC patients who underwent IMRT did not appear to benefit from concurrent chemotherapy (HR = 0.89, 95% CI 0.33–2.41, P = 0.816; heterogeneity: P = 0.046, I^2^ = 62.4%). Data regarding LRRFS were reported in four trials with 939 patients. The addition of concurrent chemotherapy led to no benefit for patients who received IMRT (P = 0.498), with HR of 1.03 (95% CI 0.95–1.12) based on a fixed-effects model, since there was no obvious evidence of heterogeneity (P = 0.759, I^2^ = 0.0%) among the included papers (Fig 2B).

A sensitivity analysis was performed to identify whether the survival results were sharply influenced by certain trials. As showed in Table 2, the survival outcomes remained stable after separately excluding three trials that recruited less than 100 patients[29–31], three low-quality studies[25, 30, 31], one trial with the median follow-up time less than 36 months[31], and two trials that enrolled a small number of stage I or III NPC patients[27, 31]. Considering that the weight of one study[26] was over 97% for the pooled result of LRRFS, we excluded this study and analyzed the residual trials. The merge HR was 0.966 (95% CI 0.559–1.667, P = 0.90; heterogeneity: P = 0.570, I^2^ = 0.0%), drawing a similar conclusion as the primary (HR = 1.03, 95% CI 0.95–1.12, P = 0.498). Generally speaking, the survival results of CCRT versus IMRT alone were of high stability.

**Table 2:** Sensitivity analysis for the comparison of CCRT with IMRT alone.

Outcome	Patients		Effect		Heterogeneity			
	CCRT	IMRT alone	HR(95% CI)	P-value	X^2^	df	I^2^(%)	P-value
Sample size > 100 patients								
OS	588	475	1.442(0.874, 2.380)	0.152	0.22	3	0	0.975
PFS	204	128	1.128(0.731, 1.740)	0.586	0.65	1	0	0.42
DMFS	384	347	1.676(0.870, 3.226)	0.122	0.3	1	0	0.581
LRRFS	464	389	1.029(0.948, 1.116)	0.493	1.14	2	0	0.564
High-quality studies								
OS	507	432	1.368(0.833, 2.245)	0.215	1.3	3	0	0.729
PFS	123	85	1.185(0.718, 1.957)	0.507	0.65	1	0	0.421
DMFS	427	390	1.498(0.809, 2.771)	0.198	1.26	2	0	0.531
LRRFS	507	432	1.029(0.948, 1.116)	0.498	1.18	3	0	0.759
Median follow-up time > 36 months								
OS	631	518	1.366(0.836, 2.231)	0.213	1.3	4	0	0.861
PFS	288	214	1.098(0.734, 1.643)	0.649	1.21	3	0	0.75
DMFS	427	390	1.498(0.809, 2.771)	0.198	1.26	2	0	0.531
LRRFS	507	432	1.029(0.948, 1.116)	0.498	1.18	3	0	0.759
Studies enrolling absolutely stage II NPC patients								
OS	390	277	1.314(0.675, 2.559)	0.442	1.27	3	0	0.736
PFS	288	214	1.098(0.734, 1.643)	0.649	1.21	3	0	0.75
DMFS	186	149	1.557(0.625, 3.882)	0.342	1.25	1	20.1	0.263
LRRFS	266	191	1.026(0.945, 1.114)	0.536	0.95	2	0	0.621

Abbreviations: CCRT, concurrent chemoradiotherapy; IMRT, intensity-modulated radiotherapy; HR, hazard ratio; CI, confidence interval; df, degrees of freedom; OS, overall survival; PFS, progression-free survival; DMFS, distant metastasis-free survival; LRRFS, locoregional recurrence-free survival.

**Fig 4:**
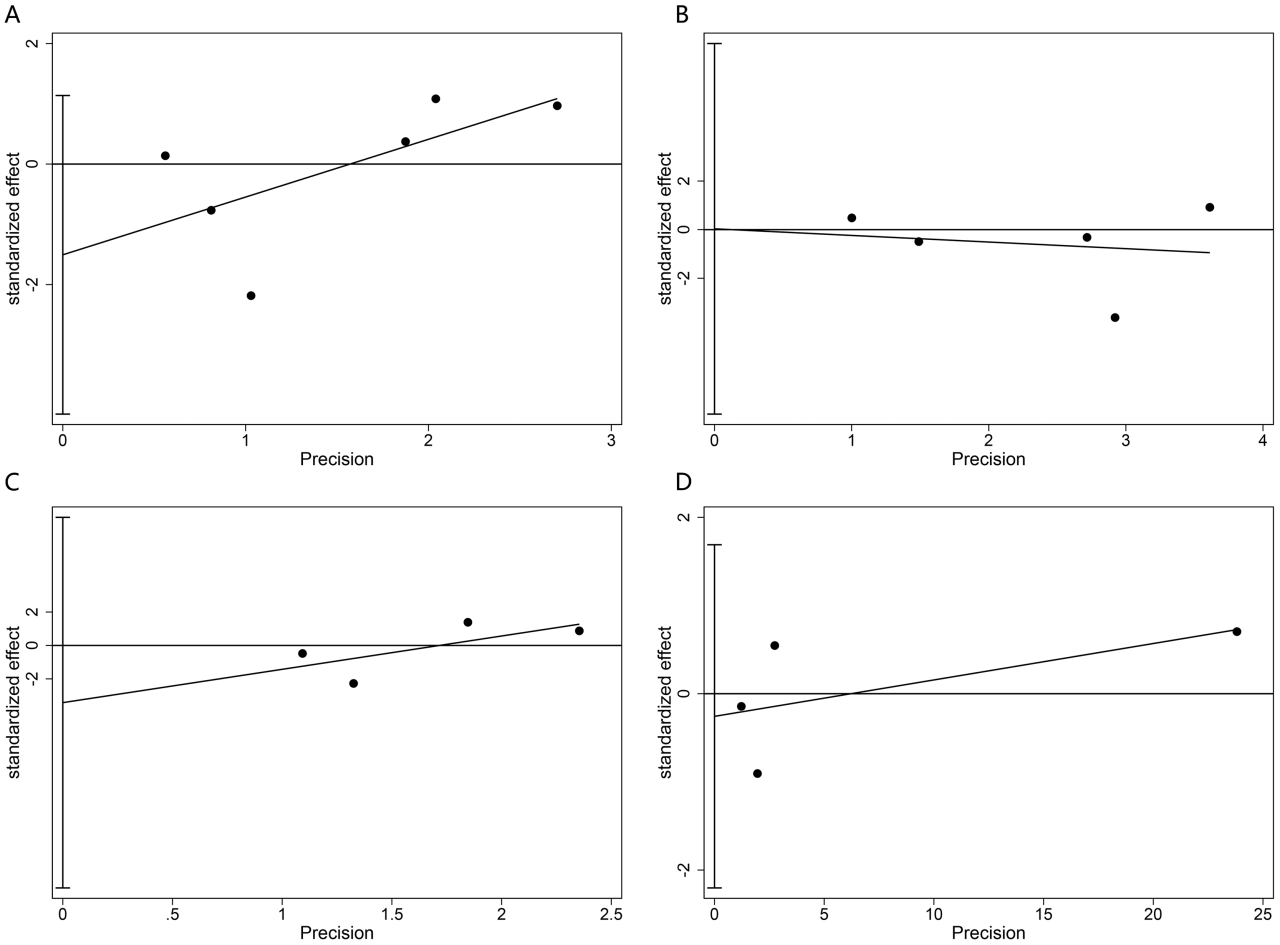
Egger’s tests for possible publication bias of OS (A), PFS (B), DMFS (C), LRRFS (D).

Both Begg’s and Egger’s tests (Fig 4) were performed, and no obvious publication bias was observed in OS, PFS, DMFS, or LRRFS (Begg’s tests, P = 0.260, 1.000, 0.734, 0.734, respectively; Egger’s tests, P = 0.189, 0.989, 0.316, 0.627, respectively).

### Treatment-related adverse events

The grade 3–4 acute adverse events that were available for pooled analysis are presented in Table 3. Compared with IMRT alone, concurrent chemoradiotherapy notably increased the risk of acute grade 3–4 leukopenia (OR = 4.432, 95% CI 2.195–8.952, P < 0.001). No significant difference was observed between the two arms with regard to the incidence of anemia (OR = 1.378, 95% CI 0.418–4.538, P = 0.598), neutropenia (OR = 1.905, 95% CI 0.801–4.529, P = 0.145), thrombocytopenia (OR = 1.981, 95% CI 0.794–4.944, P = 0.143), gastrointestinal complications (OR = 7.038, 95% CI 0.890–55.640, P = 0.064), or mucositis (OR = 1.578, 95% CI 0.949–2.623, P = 0.079).

**Table 3:** Grade 3–4 acute adverse events of CCRT versus IMRT alone for stage II nasopharyngeal carcinoma.

Grade 3–4 acute adverse events	Availability		Effect		Heterogeneity			
	CCRT (events/total)	IMRT alone (events/total)	OR (95% CI)	P-value	X^2^	df	I^2^(%)	P-value
Anemia	7/464	4/389	1.378(0.418, 4.538)	0.598	0.38	2	0	0.827
Leukopenia	49/507	10/432	4.432(2.195, 8.952)	<0.001	1.91	3	0	0.591
Neutropenia	17/321	8/283	1.905(0.801, 4.529)	0.145	0.14	1	0	0.713
Thrombocytopenia	15/507	6/432	1.981(0.794, 4.944)	0.143	3.11	3	3.6	0.375
Gastrointestinal	10/223	0/148	7.038(0.890, 55.640)	0.064	0.05	1	0	0.815
Mucositis	58/186	36/149	1.578(0.949, 2.623)	0.079	1.71	1	41.4	0.192

Abbreviations: CCRT, concurrent chemoradiotherapy; IMRT, intensity modulated radiotherapy; OR, odd ratio; CI, confidence interval; df, degrees of freedom.

## Discussion

At present, the most acceptable treatment modality for stage II NPC patients is CCRT, and the majority of evidence for this is based on conventional radiotherapy. In this systematic review and meta-analysis, we explored the real role of concurrent chemotherapy for early stage NPC patients in the IMRT era.

The results showed that the addition of concurrent chemotherapy led to comparable survival conditions for stage II NPC patients. There are several possible explanations for the non-significant survival difference. First and foremost, IMRT is obviously superior to 2D-RT in local tumor control, especially for early T-stage cases[8–11]. A retrospective study by Lai et al.[10] reported significantly improved 5-year local relapse-free survival (LRFS) (92.7% vs. 86.8%) for NPC patients treated with IMRT compared to 2D-RT, and the improvement was even greater for stage T1 patients (100% vs. 94.4%; P = 0.016). Peng et al. conducted a prospective randomized study[32] to compare clinical outcomes of IMRT versus 2D-RT for the treatment of NPC. With a median follow-up time of 42 months, the 5-year OS and local control rates were 79.6% and 90.5% for the IMRT group, and 67.1% and 84.7% for the 2D-RT group, respectively. In addition, in the study by Zhang et al.[27], NPC patients who received IMRT alone had similar survival rates with patients who received concurrent chemotherapy and 2D-RT in the study by Chen et al.[5] (4-year OS, 97.4% vs. 97.4%; 4-year DMFS, 96.5% vs. 97.3%; 4-year LRFS, 93.8% vs. 95.7%, respectively). We wonder that stage II NPC patients might not have a survival benefit from concurrent chemotherapy because IMRT is able to improve significantly the local control rate. Next, the single prospective study[5] to date that demonstrated the value of concurrent chemotherapy in the 2D-RT era enrolled stage II NPC patients evaluated using the Chinese 1992 staging system. According to the 2010 UICC/AJCC staging system, 31 of the included patients were restaged as N2 and stage III. The OS results might be falsely affected by the survival benefit from concurrent chemotherapy in these stage N2 patients. Lastly, clinical stage II NPC consisted of three subgroups-T2N0M0, T1N1M0, and T2N1M0-with different prognoses: T2b classification has a relatively greater risk of local recurrence, and T2N1 NPC might have a greater risk of distant metastasis and poorer survival[5, 13, 31, 33, 34]. Hence, we considered whether T2N1 NPC patients treated with IMRT could benefit from concurrent chemotherapy. Due to a lack of detailed data of individual patients, a subgroup assessment of stage II NPC with precise population stratification was not performed.

The pooled analysis showed that the incidence of acute grade 3–4 toxicity reactions in the CCRT group was higher than in the IMRT alone group. In particular the incidence of leukopenia reached statistical significance (OR = 4.432, 95% CI 2.195–8.952, P < 0.001). A meta-analysis by Zhang et al.[35] analyzed the overall risk of treatment-related mortality with additional chemotherapy in NPC. Compared to radiotherapy alone, chemoradiotherapy significantly increased the risk of treatment-related mortality (0.8% vs. 1.7%). Considering the increased risk of adverse events and treatment-related mortality, the management of stage II NPC should be considered with caution. At present, several phase II-III trials are ongoing to evaluate the role of CCRT for stage II NPC patients treated with IMRT (e.g., NCT02610010, NCT02116231), and we are looking forward to the eventual conclusions.

This systematic review and meta-analysis had several limitations. First, most of the included trials were retrospective, which made biases inevitable. Second, only three trials[27, 29, 31] reported survival data as HRs and 95% CIs directly. We acquired these values for the remaining trials using Tierney’s methods, which could cause potential biases and errors. Third, all of the included studies were performed in China, which might be attributed to the epidemiological characteristics of NPC. Undeniably, the generalization of the conclusions has to be carefully considered. Additionally, not all of the included studies provided sufficient data for analysis, and there was insufficient evidence of late adverse reactions to perform a pooled analysis. Despite these drawbacks, this meta-analysis may provide some significant guidance and reference to identify the optimal treatment strategy for stage II NPC patients.

## Conclusions

In brief, the present study suggested that the addition of concurrent chemotherapy led to no survival benefit and increased acute toxicity reactions for stage II NPC who received IMRT. Because patients with T2N1 have a relatively greater risk of distant metastasis, the role of adding concurrent chemotherapy to IMRT for these cases requires further research. Prospective, randomized controlled clinical trials with large sample sizes are needed.

## Supporting information

S1 File PRISMA 2009 checklist. (DOC)
